# Joint Influence of Individual Choices, Parenting Practices, and Physician Advice on Adolescent Obesity, Nebraska, 2008

**DOI:** 10.5888/pcd11.140210

**Published:** 2014-10-09

**Authors:** Hongmei Wang, Jungyoon Kim, Dejun Su, Liyan Xu, Li-Wu Chen, Terry T-K. Huang

**Affiliations:** Author Affiliations: Jungyoon Kim, Dejun Su, Li-Wu Chen, University of Nebraska Medical Center, Omaha, Nebraska; Liyan Xu, Creighton University, Omaha, Nebraska; Terry T-K Huang, City University of New York School of Public Health, New York, New York. At the time this article was written, Drs. Xu and Huang were affiliated with the University of Nebraska Medical Center, Omaha, Nebraska.

## Abstract

**Introduction:**

Reducing childhood obesity remains a public health priority given its high prevalence and its association with increased risk of adult obesity and chronic diseases. The objective of this study was to examine the joint influence of multiple risk factors on adolescent overweight status.

**Methods:**

We conducted a random-digit-dialed telephone survey of adolescents aged 12 to 19 years in fall 2008 in a Midwestern city in Nebraska. On the basis of survey data for 791 youths aged 12 to 18 years, we conducted latent class analysis to group youths by the joint occurrence of dietary behavior, physical activity, parenting practices, and physician advice. We then examined the association between the groups and overweight status by using logistic regression, controlling for age, sex, race/ethnicity, and parent and family information.

**Results:**

Youths were clustered into 3 groups. Group I (52%) were youths with healthy dietary behavior and physical activity, less permissive parenting practices, and physician advice; Group II (30%) were youths with moderately healthy dietary behavior and physical activity, less permissive parenting practices, and no physician advice; and Group III (18%) were youths with unhealthy dietary behavior and physical activity, permissive parenting practices, and physician advice. Youths in Groups I and II were less likely to be overweight than youths in Group III.

**Conclusions:**

Youths with healthier behavior and less permissive parenting practices were less likely to be overweight. Study findings highlight the need to address obesity risk factors among youths with unhealthy dietary behavior, inadequate exercise, permissive parenting practices, and some physician advice. Tailored interventions should be used to target youths with different obesity risk factors.

## Introduction

The prevalence of childhood obesity and overweight has been increasing in the United States since the 1980s. This trend appears to be stabilizing but remains high (1,2). Adolescent obesity has been particularly difficult to address: although the obesity rate for children aged 2 to 5 years has recently declined, a similar decline among children aged 6 years or older has not been seen (2). In 2012 about 21% of US youths aged 12 to 19 years were obese (2).

Childhood obesity appears to be a result of low energy expenditure and high energy intake (3). Childhood obesity interventions have focused on promoting child behavior changes for a more healthful diet, a higher level of physical activity, or both. A family-based approach is increasingly emphasized to control childhood obesity, because parents play a critical role in providing a home environment that fosters healthful eating and physical activity for children and adolescents, ultimately affecting their weight status (4,5). Recently, attention has also been given to the role of physicians in adolescents’ weight management (6). Because behavior risks are interrelated, children and adolescents who are at risk for unhealthy eating are also more likely to have less physical activity and more screen time (7). A multiple health behavior change approach for obesity prevention is needed to address multiple risk behavior domains at multiple levels (8,9).

Interest is increasing in the interconnections among multiple overweight and obesity risk factors and in tailoring interventions for different population subgroups. However, studies to date have focused on identifying the main effects of single risk factors and, to a lesser extent, of 2- or 3-way interactions. For dietary behaviors, studies showed an inconsistent relationship between fruit and vegetable consumption and child body mass index (kg/m^2^) (BMI) (10,11) and a negative association between regular breakfast consumption and childhood obesity outcomes (12,13). Studies of child physical activity and weight status also showed mixed results, although most studies found an inverse association of physical activity or sports team participation with overweight and obesity outcomes or a direct association of inactivity and sedentary behavior with overweight and obesity outcomes (14–17). Research using various measures of parenting practices regarding diet and physical activity yields inconsistent results for adolescents. One measure of parenting practice, letting a youth decide what to eat, was found to be associated with unhealthy eating behaviors, such as skipping breakfast (18). Although 1 study found that rules restricting sedentary time were associated with less TV watching (19), another study found that such rules were associated with undesirable behaviors and increases in children’s BMI (20). An authoritative parenting style, where parents are both responsive to children’s needs and controlling of children’s behaviors, was associated with healthier eating, more physical activity, and lower BMI among children (21). Physician advice has a positive effect on adolescents’ weight loss attempts (22), but no evidence has been reported on its association with adolescents’ weight change. Some studies included both physical activity and dietary behavior indicators in their analysis but did not show a consistent pattern of how these behaviors simultaneously affect adolescent obesity outcomes (23–25). To date, studies rarely considered the combination of individual behavior risk factors with parenting practices and access to physician advice, and the patterns in which these factors congregate, possibly because of methodological restrictions and unavailable data. The objective of this study was to examine the joint influence of multiple risk factors on adolescent overweight status.

We used latent class analysis (LCA) to categorize youths aged 12 to 18 years in a Midwestern city into several mutually exclusive groups based on patterns of modifiable obesity risk factors in 4 domains: dietary behavior, physical activity, parenting practices, and physician advice. To examine the joint influence of multiple risk factors on adolescent overweight, we also conducted logistic regression to see whether each of the 4 risk-factor groups was associated with overweight status, after controlling for each youth’s sociodemographic factors. The study contributes to the field in 3 ways. First, the study enhances the understanding of the unique patterns of adolescent dietary and physical activity behavior as well as parenting practices and physician involvement. Second, the study provides valuable information for stakeholders to use in prioritizing interventions and developing programs for youths with different behavior patterns. Tailored interventions for youths exposed to particular sets of risk factors use scarce health care resources more effectively. Third, this study addresses a lack of data on adolescent risk factors for obesity, especially data on parenting practices and physician advice, by providing relevant evidence to assess the importance of these factors in youths’ overweight status and to inform interventions in this high-risk group.

## Methods

### Data and sample

In a city in Nebraska, a telephone survey was conducted in September and October 2008 to collect information on adolescents’ height and weight, dietary and physical activity behavior, parenting practices, and physician advice regarding dietary behavior and physical activity. Questions measuring behavior were adapted from the Centers for Disease Control and Prevention’s (CDC’s) Youth Risk Behavior Survey Questionnaire (26). The dietary behavior and physical activity questions had moderate reliability scores (27). We administered a cross-sectional, random-digit-dialed telephone survey to youths aged 12 to 19 years who lived with their parents or guardians. Interviewers spoke first with parents or guardians to explain the study and obtain their permission for their children to participate. The interviewer then explained the study purpose and obtained the youth’s consent. If more than 1 youth aged 12 to 19 years resided in the household, 1 youth was randomly selected. Interviews were conducted in either English or Spanish. The study was approved by University of Nebraska Medical Center Institutional Review Board in 2008. We had 894 completed surveys, and a response rate of 37.5%. We excluded incomplete records and youths aged 19 years because they were considered to be young adults. Our final sample was 791 participants aged 12 to 18 years.

### Measurements

Participant overweight status was determined by whether the youth’s BMI percentile was equal to or greater than the 85th percentile for his or her age and sex based on the standard CDC growth chart.

Nine observed items in the following 4 domains were used for LCA to identify subgroups of youths: 1) Dietary behavior was captured by 2 variables: whether the youth a) ate breakfast every day and b) consumed at least 5 servings of fruits and vegetables on 4 or more days a week. 2) Physical activity was measured by 3 variables: a) whether the youth engaged in at least 1 hour of physical activity on 4 or more days a week, b) participated on at least 1 sports team, and c) watched television or played video games less than 2 hours a day during weekdays. 3) Parenting practices regarding eating and physical activity were measured by 2 variables: whether parents let the youth decide a) what to eat and b) how long to watch television or play video games. 4) Physician advice was measured by 2 variables: whether, during the last routine physical examination within the previous 12 months, a doctor talked with the youth about a) nutrition and healthy eating and b) exercise and physical activity.

Age was categorized into 2 groups: 12 to 14 years and 15 to 18 years. Race/ethnicity was considered a binary indicator with non-Hispanic white as the reference group. Family structure was used as a binary indicator with youths not living with both parents as the reference group. The indicator for parents’ education was whether either parent attended college or university.

### Analysis

We used LCA to create mutually exclusive subgroups of adolescents. LCA is a statistical technique to classify subjects into smaller subgroups on the basis of their response patterns to a set of observed categorical variables (28). LCA uses maximum likelihood estimation to obtain latent class membership probabilities and item-response probabilities. An item-response probability that is close to 1 or 0 indicates greater group heterogeneity. We examined multiple models to fit different numbers of latent classes and chose the best model by using the Bayesian information criterion (BIC). A lower BIC implies a better balance between fitting the data and parsimony (28,29). After we classified the participants using LCA, we examined the differences among groups and ran a weighted logistic regression to examine the relationship between group members and each youth’s overweight status. SAS version 9.2 (SAS Institute, Inc) and Proc LCA version 1.2.5 (Penn State Methodology Center) were used for analysis.

## Results

Forty-six percent of participants were aged 12 to 14 years, 53% were boys, 85% were non-Hispanic white, 12% had no parent who attended college or university, and 87% lived with both parents (Table 1).

**Table 1:** Demographic and Behavioral Characteristics of Surveyed Youths, Total Sample and by Group, Nebraska, 2008

Variables	Total Sample (n = 791), %	Group I (n = 410), %	Group II (n = 237), %	Group III (n = 144), %
Percentage of total sample	100	52	30	18
**Age, y**				
12–14	46	56^b^^,^ c	36^a^	35^a^
**Sex**				
Male	53	57^c^	53	41^a^
**Race/ethnicity**				
Non-Hispanic white	85	89^c^	85	76^a^
**Parents’ education**				
Neither parent went to college	12	9^c^	12	19^a^
**Family structure**				
Lives with both parents	87	91^b^^,^ c	82^a^	83^a^
**Dietary behaviors**				
Eats breakfast every day	61	76^b^^,^ c	55^a^^,^ c	25^a^^,^ b
Eats 5 servings of fruits and vegetables a day for 4 days or more per week	63	75^b^^,^ c	55^a^	44^a^
**Physical activities**				
Has 60 min of physical activity on 4 days or more per week	80	92^b^^,^ c	80^a^^,^ c	47^a^^,^ b
Participates on at least 1 sports team	77	93^b^^,^ c	73^a^^,^ c	37^a^^,^ b
Watches TV or plays video games less than 2 h per day on weekdays	67	78^b^^,^ c	65^a^^,^ c	39^a^^,^ b
**Parenting practices**				
Parents let youth choose what to eat	80	75^c^	81^c^	94^a^^,^ b
Parents let youth decide how much to watch TV or play video games	66	61^c^	62^c^	88^a^^,^ b
**Physician advice**				
Physician mentioned nutrition during last routine physical exam	57	86^b^^,^ c	0^a^^,^ c	67^a^^,^ b
Physician mentioned physical activity during last routine physical exam	61	88^b^	0^a^^,^ c	85^b^
**Overweight status**				
BMI (kg/m^2^) equal to or greater than 85th percentile	24	21^c^	20^c^	41^a^^,^ b

Abbreviations: BMI, body mass index.

a Indicates the number is significantly different from that of Group I at the *P* = .05 level.

b Indicates the number is significantly different from that of Group II at the *P* = .05 level.

c Indicates the number is significantly different from that of Group III at the *P* = .05 level.

Over 61% reported eating breakfast every day, and 63% reported eating 5 or more servings of fruits and vegetables per day for 4 days or more a week. Eighty percent engaged in at least 60 minutes of physical activity for 4 days or more a week, 77% participated in at least 1 sports team, and 67% watched television or played video games for less than 2 hours a day during weekdays. Eighty percent of participants reported that their parents let them choose what to eat, and 66% reported that their parents let them decide how much time to spend watching television or playing video games. During their last routine physical examination within the previous12 months, 57% received physician advice on nutrition and 61% received physician advice on physical activity. About 24% were overweight, which is lower than the national average (2).

### Latent class analysis results

Three unique latent classes emerged (Table 2). Group I showed healthy dietary behavior, with the highest probability of eating breakfast every day (0.76) and of having 5 servings of fruits and vegetables for 4 or more days a week (0.76). Youths in this group also engaged in the most physical activity (the probability of having 1 hour of physical activity at least 4 days a week was 0.90, of participating on at least 1 sports team was 0.92, and of having less than 2 hours of screen time was 0.78). Of the 3 groups, youths in Group I were most likely to have received physician advice during the last routine physical examination (advice on nutrition, 0.84; advice on physical activity, 0.86). Parents in this group were less permissive regarding television watching or video game playing (the probability of letting adolescents decide how much time to watch television or play video games was 0.60). Group I consisted of 52% of the sample.

**Table 2:** Latent Classes of Adolescents Based on Dietary Behavior, Physical Activity, Parenting Practices, and Physician Advice (N = 791)^a^, Nebraska, 2008

Response Probability	Group I	Group II	Group III
	Healthy Dietary Behavior and Physical Activity, Less Permissive Parenting, and Physician Advice	Moderately Healthy Dietary Behavior and Physical Activity, Less Permissive Parenting, No Physician Advice	Unhealthy Dietary Behavior and Physical Activity, More Permissive Parenting, and Physician Advice
**Dietary behaviors**			
Eats breakfast every day	0.76	0.55	0.32
Eats 5 servings of fruits and vegetables on 4 or more days a week	0.76	0.54	0.45
**Physical activities**			
Has 60 min of physical activity on 4 or more days a week	0.90	0.80	0.56
Participates on at least 1 sports team	0.92	0.73	0.47
Watches television or plays video games less than 2 h per day during weekdays	0.78	0.63	0.42
**Parenting practices**			
Parents let youth choose what to eat	0.74	0.81	0.93
Parents let youth decide how much to watch television or play video games	0.60	0.62	0.86
**Physician advice**			
Physician mentioned nutrition at last routine physical exam	0.84	0.01	0.67
Physician mentioned exercise or physical activity at last routine physical exam	0.86	0.04	0.79

a A 3-group model showed the best fit indices (Akaike information criterion = 531.5; Bayesian information criterion = 667.1; G^2^ = 473.5; degrees of freedom = 482).

Group II youths had unhealthier dietary behavior and less physical activity, more permissive parenting practices, and lower physician involvement than those in Group I. Adolescents in this group still showed a high probability of physical activity behavior (eg, involved in at least 1 sports team, 0.73), but had a moderate probability of having healthy dietary behavior (eg, eating breakfast every day, 0.55). Probabilities for permissive parenting practices were higher than in Group I (eg, letting the youth choose what to eat, 0.81, and how much time to watch television and play video games, 0.62). A unique characteristic of Group II was that it had the lowest probabilities of receiving physician advice (advice on nutrition, 0.01, and advice on physical activity, 0.04). This group consisted of 30% of the adolescents in the sample.

Group III had the unhealthiest dietary behavior and physical activity and the most permissive parenting practices. Members of this group were less likely to report healthy dietary behavior (eg, fruit and vegetable consumption, 0.45) and physical activity (eg, less screen time, 0.42). Probabilities for permissive parenting practices for this group were the highest among the 3 groups (0.93 and 0.86). Youths in this group were also more likely to receive physician advice (0.67 and 0.79) than those in Group II. This group consisted of 18% of the youths in the sample.

Compared with youths in Group I, youths in Group III were more likely to be overweight, older, female, and from a minority race or ethnic group; were more likely to have non-college educated parents; and were less likely to live with both parents (Table 1). Youths in Group II were also older and less likely to live with both parents than those in Group I.

###  Logistic regression results

The logistic regression results suggest that Group I youths were less likely to be overweight than those in Group III, the reference group (odds ratio [OR], 0.44; 95% confidence interval [CI], 0.26–0.75) (Table 3). Similarly, youths in Group II were also less likely to be overweight than those in Group III (OR, 0.35; 95% CI 0.25–0.48). The comparison between these groups suggested that healthy individual behaviors and less permissive parenting practices regarding physical inactivity were conducive to healthier weight status among adolescents.

**Table 3:** Logistic Regression Results of Predicting Youth Overweight Status by Youth Groups after Controlling for Demographic Variables (N = 791), Nebraska, 2008

Explanatory Variables	Odds Ratio (95%CI)	*P* Value
Group I	0.44(0.26–0.75)	.002
Group II	0.35(0.25–0.48)	<.001
Group III	1 [Reference]	
Aged 12–14 y	1.21(0.89–1.67)	0.23
Aged 15–19 y	1 [Reference]	
Male	1.50(1.42–1.59)	<.001
Female	1 [Reference]	
Non-Hispanic white	0.31(0.22- 0.46)	<.001
Minority race^a^	1 [Reference]	
Neither parent attended college or university	1.12(0.84–1.49)	0.44
Either parent attended college or university (reference group)	1 [Reference]	
Lives with both parents	0.87(0.56–1.32)	0.51
Does not live with both parents	1 [Reference]	

Abbreviation: CI, confidence interval.

a Includes all youths who did not identify themselves as non-Hispanic white.

The logistic regression results suggested that boys were more likely to be overweight than girls (OR, 1.50; 95% CI, 1.42–1. 59). Non-Hispanic white youths were less likely to be overweight than youths from minority races (OR, 0.31; 95% CI, 0.22–0.46). Parents’ education, youth’s age, and youth living with both parents were not significantly associated with overweight status.

## Discussion

Our results indicated that adolescents in our study were differently exposed to the 4 obesity risk factors we considered: dietary behavior, physical activity, parenting practices, and physician advice. Three latent classes emerged: youths with healthy dietary behavior and physical activity, less permissive parenting practices, and physician advice (Group I); youths with moderately healthy dietary behavior and physical activity, less permissive parenting practices, and physician advice (Group II); and youths with unhealthy dietary behavior and physical activity, permissive parenting practices, and physician advice (Group III). The group patterns identified in our analysis suggest that unhealthy behaviors are not only interrelated, as suggested in the literature (7); they also co-occur with permissive parenting practices and physician advice to youths.

The logistic regression analysis results suggested that categorizing these youths into 3 groups was meaningful in explaining youth weight status. Youths from Group III were more likely to be overweight than those from Group I and II. This finding highlights the need to make addressing obesity risk factors a priority for adolescents with unhealthy eating behaviors, inadequate exercise, more permissive parenting practices, and physician advice. Compared with youths in Group I, youths in Group III were likely to be older, female, and from a minority race; to have non-college educated parents; and less likely to live with both parents. Interventions targeting this group should be the priority for allocating limited resources to address adolescent obesity and overweight. This finding also supports the call for interventions to target multiple risk behaviors that address multiple domains of obesity risk factors (8). Multidimensional interventions in all 4 areas should be jointly implemented to improve the weight status of youths in Group III.

This study adds to the current understanding of adolescent obesity by illustrating how risk factors jointly influence the weight status of youths. Healthy individual behaviors and less permissive parenting practices together had a strong association with a better weight outcome for adolescents. Because parents in Group I and II were less permissive regarding sedentary behavior than parents in Group III, our results reinforce previous findings that some parental control over behaviors may be effective in maintaining healthy weight among youths (18,19). A family-based approach that considers family background and stresses the role of parenting in shaping children’s obesity-related behaviors may be most effective (4,5).

Interestingly, we did not observe much difference in weight status between Group I and Group II. Although youths in both groups were physically active and received similar parenting, youths in Group II showed less healthy dietary behavior and were less likely to receive physician advice on nutrition and physical activity than those in Group I. One possible explanation for the lack of difference between these 2 groups might be the lack of specificity in some behavior indicators, such as total calorie or total sugar and fat intake (11). The results could be due to Group II having an elevated risk for obesity but having not yet gained excessive weight. If this explanation is accurate, then this group should also be targeted for future intervention to prevent obesity. Another plausible explanation could be that a physician did not discuss nutrition or physical activity during a consultation with healthy-weight youths. Because only about half of pediatricians calculate adolescents’ BMI during a routine visit (30), there is still room for physicians to play a more active role in obesity prevention.

This study has several limitations. First, given that the study sample was from a single Midwestern city and the response rate was relatively low, the results may not be readily generalizable to a larger population. Second, the cross-sectional data used do not allow us to infer causality, and the data did not include information on some obesity risk factors such as inadequate sleep and poor mental health status. Third, the parenting practices and physician advice measures used were relatively crude, and validated measures should be used in future studies. Lastly, we used self-reported BMI to measure overweight status, which may have underestimated the likelihood of overweight. However, we do not expect that these limitations are sufficiently severe to change study results on the youth groups and their relationship to overweight status.

These limitations notwithstanding, our study showed a need to simultaneously examine the complex set of factors and their patterned congregations in relation to adolescent obesity. Concrete efforts in individual, family, and primary care settings are important to shape adolescent behaviors and to help adolescents maintain a healthy weight. The physician’s role may need to be expanded from a predominant focus on management of obesity-related conditions to a more active approach to obesity prevention. The 3 youth groups identified in our study and their different risks of overweight also suggest that obesity interventions will be more effective if they are customized for particular behavioral and contextual phenotypes of youth.
